# Mouse Background Strain Profoundly Influences Paneth Cell Function and Intestinal Microbial Composition

**DOI:** 10.1371/journal.pone.0032403

**Published:** 2012-02-27

**Authors:** Ajay S. Gulati, Michael T. Shanahan, Janelle C. Arthur, Emily Grossniklaus, Richard J. von Furstenberg, Lieselotte Kreuk, Susan J. Henning, Christian Jobin, R. Balfour Sartor

**Affiliations:** 1 Center for Gastrointestinal Biology and Disease, University of North Carolina at Chapel Hill, Chapel Hill, North Carolina, United States of America; 2 Department of Pediatrics, Division of Gastroenterology and Hepatology, University of North Carolina at Chapel Hill, Chapel Hill, North Carolina, United States of America; 3 Department of Medicine, Division of Gastroenterology and Hepatology, University of North Carolina at Chapel Hill, Chapel Hill, North Carolina, United States of America; 4 Department of Cellular and Molecular Physiology, University of North Carolina at Chapel Hill, Chapel Hill, North Carolina, United States of America; 5 Department of Pharmacology, University of North Carolina at Chapel Hill, Chapel Hill, North Carolina, United States of America; 6 Department of Microbiology and Immunology, University of North Carolina at Chapel Hill, Chapel Hill, North Carolina, United States of America; Charité-University Medicine Berlin, Germany

## Abstract

**Background:**

Increasing evidence supports the central role of Paneth cells in maintaining intestinal host-microbial homeostasis. However, the direct impact of host genotype on Paneth cell function remains unclear. Here, we characterize key differences in Paneth cell function and intestinal microbial composition in two widely utilized, genetically distinct mouse strains (C57BL/6 and 129/SvEv). In doing so, we demonstrate critical influences of host genotype on Paneth cell activity and the enteric microbiota.

**Methodology and Principal Findings:**

Paneth cell numbers were determined by flow cytometry. Antimicrobial peptide (AMP) expression was evaluated using quantitative reverse-transcriptase polymerase chain reaction (qRT-PCR), acid urea-polyacrylamide gel electrophoresis, and mass spectrometry. Effects of mouse background on microbial composition were assessed by reciprocal colonization of germ-free mice from both background strains, followed by compositional analysis of resultant gut bacterial communities using terminal restriction fragment length polymorphism analysis and 16 S qPCR. Our results revealed that 129/SvEv mice possessed fewer Paneth cells and a divergent AMP profile relative to C57BL/6 counterparts. Novel 129/SvEv á-defensin peptides were identified, including Defa2/18v, Defa11, Defa16, and Defa18. Host genotype profoundly affected the global profile of the intestinal microbiota, while both source and host factors were found to influence specific bacterial groups. Interestingly, ileal α-defensins from 129/SvEv mice displayed attenuated antimicrobial activity against pro-inflammatory *E. coli* strains, a bacterial species found to be expanded in these animals.

**Conclusions and Significance:**

This work establishes the important impact of host genotype on Paneth cell function and the composition of the intestinal microbiota. It further identifies specific AMP and microbial alterations in two commonly used inbred mouse strains that have varying susceptibilities to a variety of disorders, ranging from obesity to intestinal inflammation. This will be critical for future studies utilizing these murine backgrounds to study the effects of Paneth cells and the intestinal microbiota on host health and disease.

## Introduction

Paneth cells are highly specialized secretory cells of the intestinal epithelium, located at the base of the crypts of Lieberkühn in the small intestine. Their distinctive morphology is characterized by large secretory granules that contain a diverse array of proteins that support intestinal homeostasis. These homeostatic functions range from maintenance of the intestinal stem cell niche [1] to establishment of the antimicrobial barrier of the intestinal mucosa [2]. The most abundant proteins found in Paneth cell granules are known as antimicrobial peptides (AMPs) [3]. In the healthy host, Paneth cells utilize AMPs to provide defense against enteric pathogens [4], [5], and to modulate the composition of the commensal gut microbiota [6]. Because increasing evidence suggests that the enteric microbiota plays a key role in regulating host physiology [7], elucidating the mechanisms that control Paneth cell function is critical to our further understanding of a wide spectrum of human disease.

Crohn's disease (CD) is a specific human clinical disorder recently linked to Paneth cell dysfunction [8], [9]. CD is a chronic, immune-mediated, inflammatory condition of the intestinal tract that is thought to result from dysregulated immune responses to the endogenous intestinal microbiota [10]. Because Paneth cells can modulate the commensal bacteria of the gut, they are in a key position to influence the pathogenic host immune responses believed to drive intestinal inflammation in patients with CD. Interestingly, CD most commonly affects the distal ileum where Paneth cell abundance is greatest [11]. In addition, numerous CD susceptibility loci have been identified, which contain genes that may influence Paneth cell activity and AMP expression. Specifically, polymorphisms of the CD risk alleles NOD2, TCF-4, ATG16L1, and XBP1 have all been associated with Paneth cell dysfunction [12]–[15]. These findings suggest a strong influence of host genotype on Paneth cell function, as well as the potential for downstream effects on the enteric microbiota.

Despite the putative impact of host genotype on Paneth cell function, direct support for this interaction has been difficult to generate. This is due, in part, to the important role that the commensal microbiota plays in regulating Paneth cell activity. Specifically, both live bacteria and their cellular components can induce Paneth cell degranulation and secretion [3]. Furthermore, certain AMPs such as angiogenin 4 (Ang4) and regenerating islet-derived protein 3 gamma (Reg3γ) are known to be transcriptionally induced by bacterial colonization of the intestine [16], [17]. Therefore, separating the effects of host genotype and the enteric microbiota on Paneth cell activity has been difficult to establish.

In the present study, we sought to assess the direct influences of host genotype on both Paneth cell function and the composition of the intestinal microbiota. To accomplish this, we comprehensively evaluated Paneth cells in two widely used inbred mouse strains, namely C57BL/6 (B6) and 129/SvEv (129) mice. Importantly, germ-free (GF) mice from both backgrounds were utilized in selected experiments to control for the potentially confounding influences of the enteric microbiota on Paneth cell function. Our results reveal key differences in Paneth cell number and AMP expression between B6 and 129 mice, as well as a strong impact of host background on the composition of the intestinal microbiota. These findings will be essential in designing future murine studies that explore physiologic and/or disease-related processes involving Paneth cells and the intestinal microbiota.

## Results

### 129 Mice Possess Fewer Paneth Cells than B6 Mice

**Figure 1:**
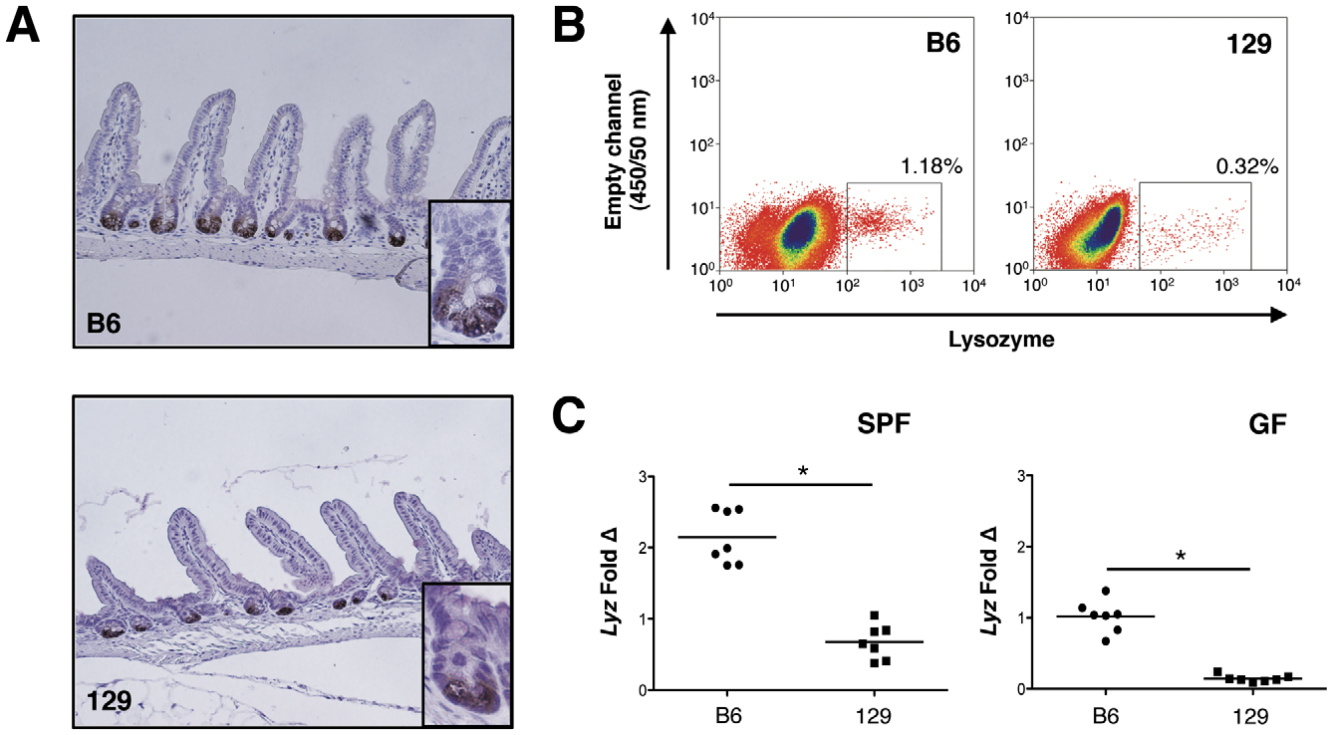
129 mice possess fewer Paneth cells than B6 mice. (A) Lysozyme (Lyz) staining of ileal tissue shown at 20× magnification from B6 and 129 mice. Inset shows high power view (40×) of single crypt. (B) Flow cytometry was used to quantify Lyz^+^ cells from the ileal epithelium of B6 and 129 mice. Data are representative of 3 independent experiments. (C) *Lyz* transcripts were measured using quantitative RT-PCR of ileal RNA extracts from SPF and GF mice. Copy number is normalized to *Gapdh*, and fold change is relative to the GF B6 group (*P<.0001).

Paneth cell numbers in the ilea of B6 and 129 mice were first assessed qualitatively using anti-Lyz immunohistochemistry (Figure 1A). Despite similar numbers of crypts and villi, there was decreased Lyz^+^ staining in the 129 mice. This was confirmed quantitatively using flow cytometry to determine the percentage of Lyz^+^ cells in ileal epithelial cell preparations from both mouse strains. As shown in Figure 1B, B6 mice possessed a higher percentage of Lyz^+^ epithelial cells than their 129 mice counterparts. Repeat analyses (n = 3) revealed B6 mice had 0.83±0.32% Lyz^+^ epithelial cells versus 0.23±0.09% in 129 mice (∼4-fold difference, *P*<.04). This was consistent with ileal mRNA expression of *Lyz* shown in Figure 1C, which demonstrated that specific pathogen-free (SPF) B6 mice expressed ∼3-fold more *Lyz* transcript than 129 mice (*P*<.0001). Interestingly, a similar pattern was observed in GF mice, with GF B6 mice expressing ∼7-fold greater *Lyz* mRNA than 129 mice (*P*<.0001). These findings demonstrate that the observed differences in Paneth cell number are not due solely to disparities in the intestinal microbial environment between the two mouse strains.

### B6 and 129 Mice Display Distinct Patterns of Ileal AMP Expression

**Figure 2:**
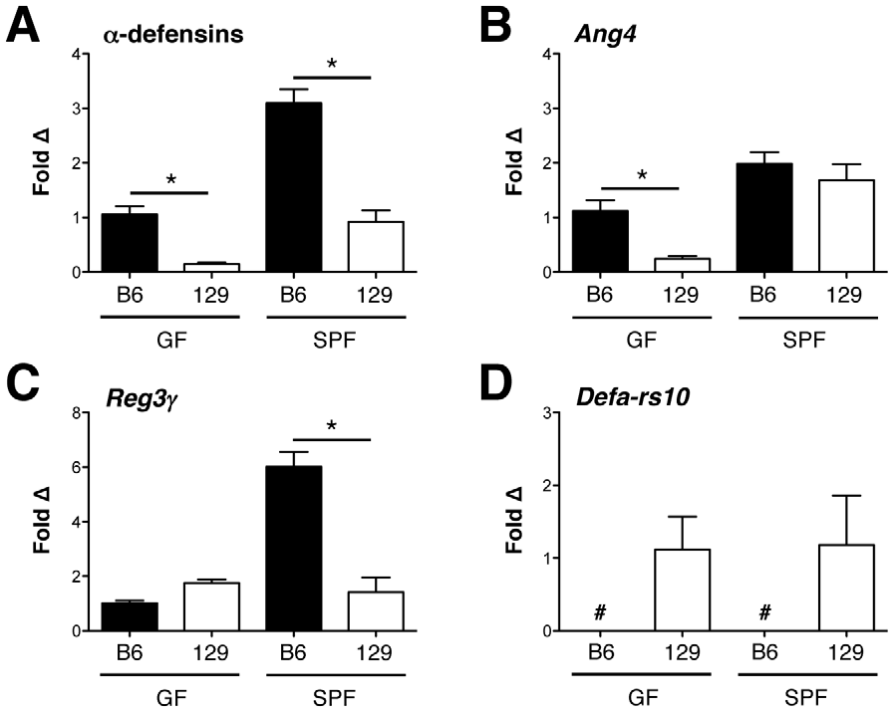
B6 and 129 mice display distinct patterns of ileal antimicrobial peptide expression. Ileal transcript levels of (A) α-defensins; (B) angiogenin 4 (*Ang4*), (C) regenerating islet-derived protein 3 gamma (*Reg3γ*); and (D) α-defensin related sequence 10 (*Defa-rs10*) are shown for B6 and 129 GF and SPF mice (n = 6–7 mice/group). Data are shown as means with SEM (*P<.05). *Defa-rs10* was not detected (#) in any B6 animal.

Given that 129 mice possessed fewer Paneth cells than their B6 counterparts, we sought to determine if this was associated with changes in the secreted products these cells utilize to control intestinal microbial populations. In addition to Lyz, these antimicrobial molecules include: α-defensins (cryptdins), cryptdin-related sequence (CRS) peptides, Ang4, and Reg3γ [18]. To measure α-defensin expression, we utilized quantitative reverse-transcriptase polymerase chain reaction (qRT-PCR) primers known to simultaneously detect all known α-defensin genes [19]; for the other AMP classes, we selected representative molecules for each group. Because microbial colonization can modulate AMP levels [20], we performed these analyses on both GF and SPF mice. Figure 2 demonstrates varying patterns of AMP mRNA expression between the 2 mouse strains. α-Defensin expression paralleled *Lyz*, with reduced levels in both GF and SPF 129 mice relative to their B6 counterparts (Fig. 2A). Expression of *Ang4* was also reduced in GF 129 mice, suggesting an effect of host genotype on levels of this AMP; however, under SPF conditions, *Ang4* levels were similar in both mouse strains, indicating that bacterial influences can over-ride the host genotype effect (Fig. 2B). Conversely, *Reg3γ*expression was similar in both mouse strains under GF conditions, but was strikingly increased in B6 versus 129 mice reared in an SPF environment. This suggests a secondary environmental or microbial effect on *Reg3γ* expression (Fig. 2C). Interestingly, a representative CRS peptide known as *Defa-rs10* was not detected in B6 mice, but present in 129 animals (Fig. 2D). This finding demonstrates that reduced Paneth cell numbers in the 129 mice cannot explain the entire spectrum of AMP differences present between these mouse strains.

### B6 and 129 Mice Display Distinct Patterns of α-Defensin Expression

**Figure 3:**
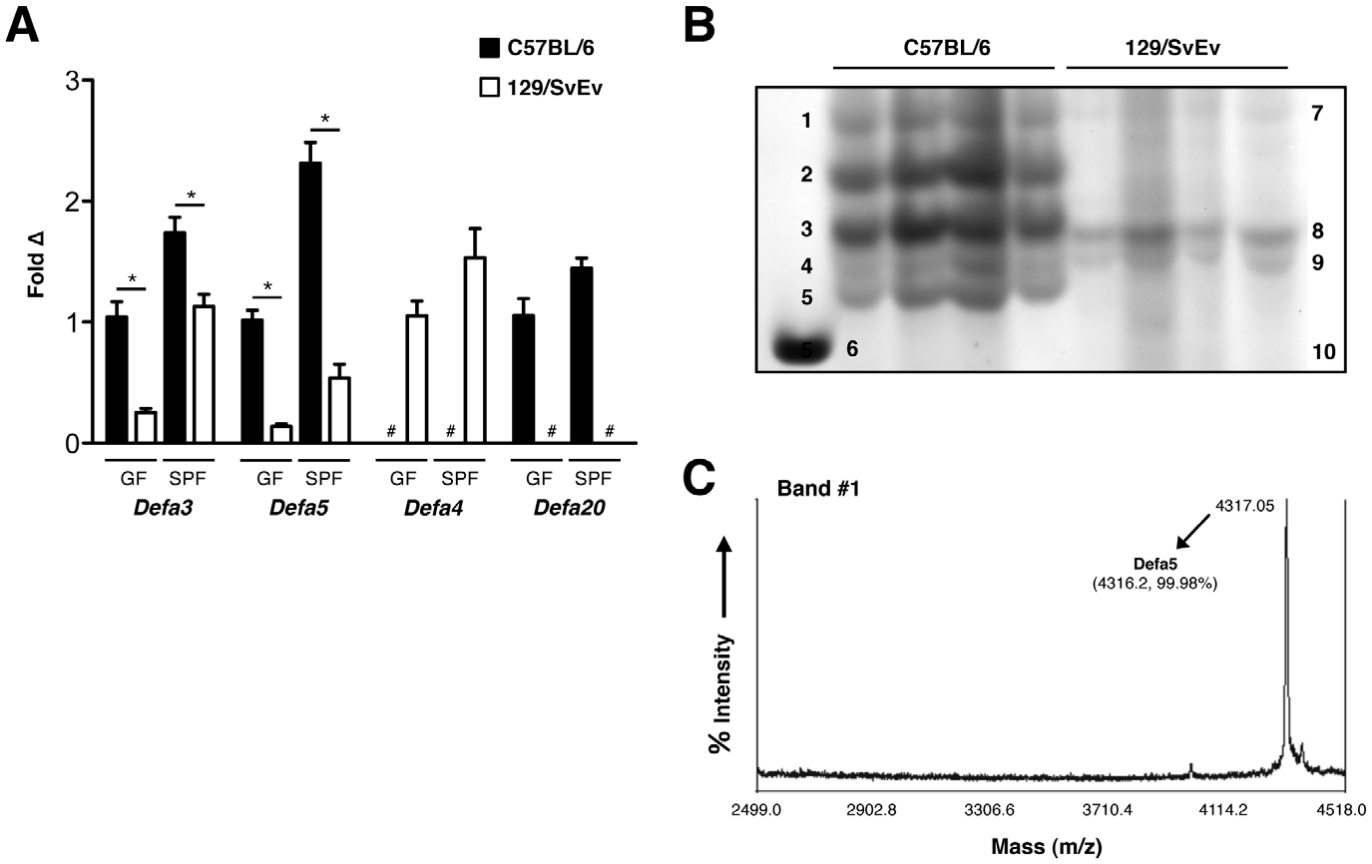
B6 and 129 mice exhibit distinct patterns of ileal α-defensin expression. (A) mRNA expression of α-defensin isoforms in the ileum of B6 and 129 GF and SPF mice (n = 7/group). Copy number is normalized to *Gapdh* (*P<.05, # - not detected). (B) AU-PAGE demonstrates peptide expression patterns of α-defensin isoforms in the ileum of B6 and 129 SPF mice. First lane is recombinant Defa4 control; each additional lane represents an individual mouse. Individual bands (based on calculated mass determined via mass spectrometry): 1-Defa5; 2-Defa24, Defa27; 3-Defa20, Defa21; 4-Defa2; 5-Defa22; 6-Defa4 (recombinant); 7-Defa5; 8-Defa2/18v, Defa11, Defa16, Defa21; 9-Defa18, Defa25; 10-Defa4. (C) Representative mass spectrum for band #1. Mass data for all gel bands are summarized in Table S1.

Because α-defensins confer the majority of antimicrobial activity in Paneth cell secretions [3], we next sought to further explore the finding that this class of AMPs is reduced in 129 mice relative to their B6 counterparts. To characterize specific α-defensin differences, we measured ileal transcript levels of α-defensins from 3 different phylogenetic groups (*Defa3*, *Defa5*, *and Defa4/20*) [21]. As shown in Figure 3A, the α-defensins *Defa3* and *Defa5* were decreased in 129 mice relative to their B6 counterparts. *Defa4* was expressed only on 129 mice, while *Defa20* was detected only in B6 animals. These differential patterns of expression were consistent in both GF and SPF mice. To determine if these disparities in α-defensin expression were present at the peptide level, acid urea-polyacrylamide gel electrophoresis (AU-PAGE) was used to assess the ileal α-defensin peptide fraction of B6 and 129 mice. As demonstrated in Figure 3B, this revealed strikingly different α-defensin protein migration patterns between the two mouse strains.

To determine the specific α-defensin peptides present in B6 and 129 mice, we next utilized matrix assisted laser desorption-time of flight tandem mass spectrometry (MALDI-TOF MS/MS) to identify the individual bands revealed in our AU-PAGE protein analysis (Fig. 3C). These data are summarized in Table S1. Findings at the peptide level were consistent with our mRNA results, but also identified additional differences in α-defensin expression. The primary α-defensin peptides in B6 mice included Defa2, Defa5, Defa20, Defa21, Defa22, Defa24, and Defa27, while Defa3 and Defa23 appeared to be minor constituents. These findings are consistent with previously published data [21]. In contrast to B6 mice, the α-defensin class has not been characterized in 129 mice at the peptide level. In 129 animals, we found the major α-defensins to include Defa2/18v, Defa4, Defa5, Defa11, Defa16, Defa21, Defa18, and Defa25. Therefore, while Defa3, Defa5, and Defa21 are shared between the two mouse strains, numerous additional α-defensins are unique to each background.

### Host Genotype Affects Composition of the Intestinal Microbiota

**Figure 4:**
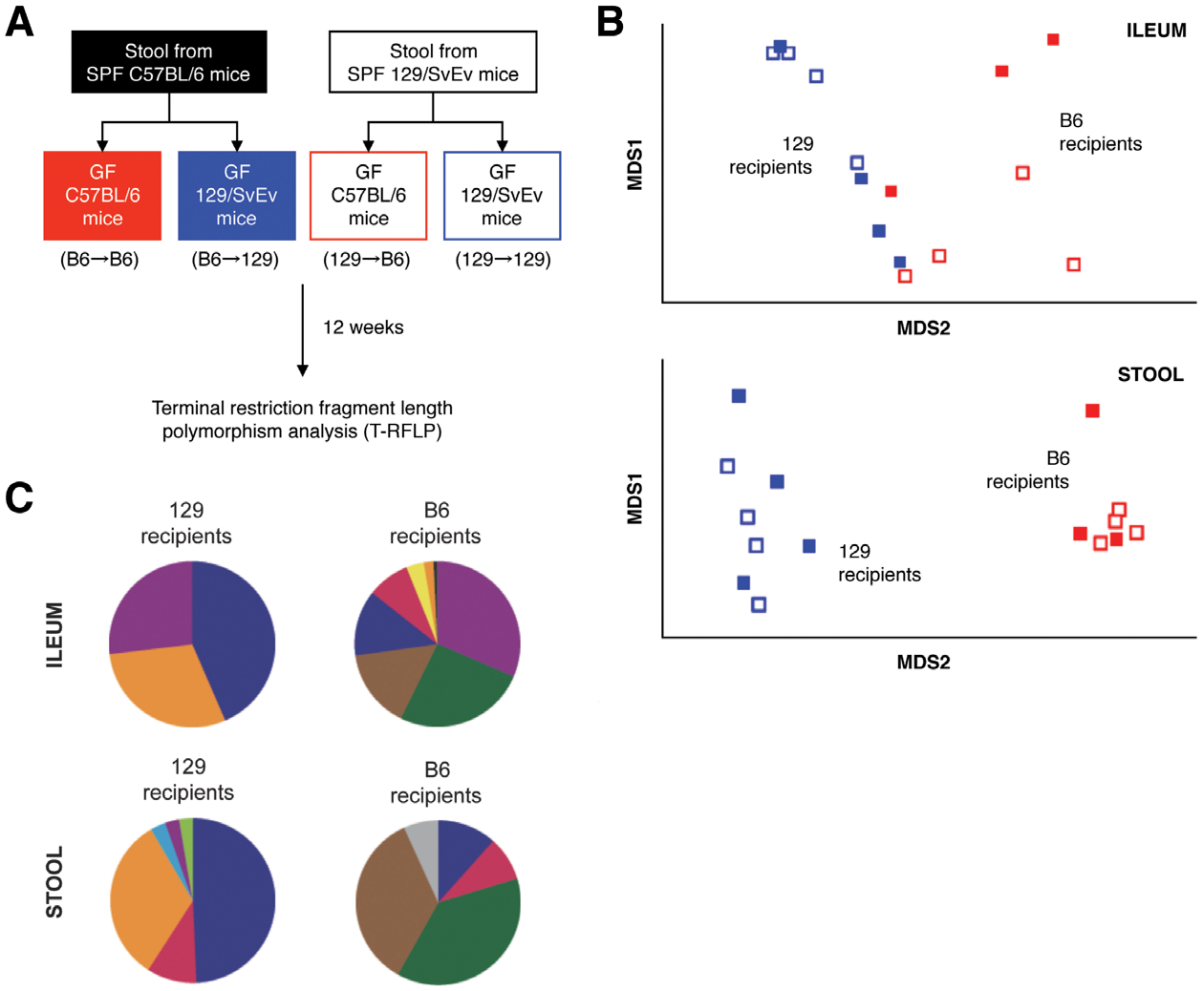
Host genotype affects the composition of intestinal microbiota. (A) 4 experimental groups were defined by colonizing GF B6 or 129 mice with the stool microbiota of SPF B6 or 129 mice (Donor→Recipient). Ileal mucosa and stool bacterial communities were analyzed by T-RFLP at 12 wk post-colonization. (B) TRFs derived from these communities were used to generate nonmetric multidimensional scaling plots for ileal tissue and stool samples. In both bacterial compartments, the 129 (blue) and B6 (red) recipient groups were statistically dissimilar based on ANOSIM analysis (*P*<.05, n = 3–4 mice/group). (C) Percent contribution of T-RFs to each group (top 100% for ILEUM, top 90% for STOOL). Each color represents a unique T-RF indicating a putative bacterial group.

Having established that B6 and 129 mice possess differences in Paneth cell number and AMP expression, we speculated that these disparities would be associated with alterations of the commensal gut microbiota within these mouse strains. To demonstrate that B6 and 129 mice possess inherently different intestinal microbial communities, we colonized GF B6 and 129 mice with identical bacterial sources, and then profiled their resultant ileal and fecal bacterial populations. As depicted in Figure 4A, stool from either SPF B6 or 129 mice was used to colonize individual cohorts of GF B6 and 129 animals, thereby creating 4 experimental groups representing all possible donor→recipient combinations (B6→B6, B6→129, 129→B6, 129→129; n = 3–4 mice/group). Bacterial community fingerprinting was performed 12 wk after colonization, using terminal restriction fragment length polymorphism (T-RFLP) analysis of the 16 S rRNA gene.

As shown in Figure 4B, bacterial communities clustered based on the background strain of the recipient mice, regardless of bacterial source (*P*<.05). This was true for the mucosally-associated bacteria of the ileum, as well as fecal bacterial communities. It is important to note that no differences were detected when clustering was performed based on bacterial source alone (*P*<.81 for ileum and *P*<.46 for stool). Figure 4C depicts the percent contribution of individual T-RFs to the total bacterial communities of 129 and B6 recipients. Because it is difficult to ascribe specific bacterial groups to a given T-RF, each color in Figure 4C represents a different putative bacterial group. Although this does not precisely describe the bacterial composition of the 2 recipient groups, it does confirm that the global structure and abundance of individual bacterial groups differed between host recipient strains. While it appears that general bacterial complexity is decreased in the ileal mucosa of 129 mice, it should be noted that the overall microbial diversity (based on the Shannon diversity index) within both the ileal mucosa and fecal samples was not significantly different between 129 and B6 mice (ileum, *P*>.23; stool, *P*>.62). In summary, these findings demonstrate that host genotype is an important driving force for the composition of the intestinal microbiota.

### Host Genotype and Bacterial Source Affect Abundances of Specific Bacterial Groups Within the Intestinal Microbiota

**Figure 5:**
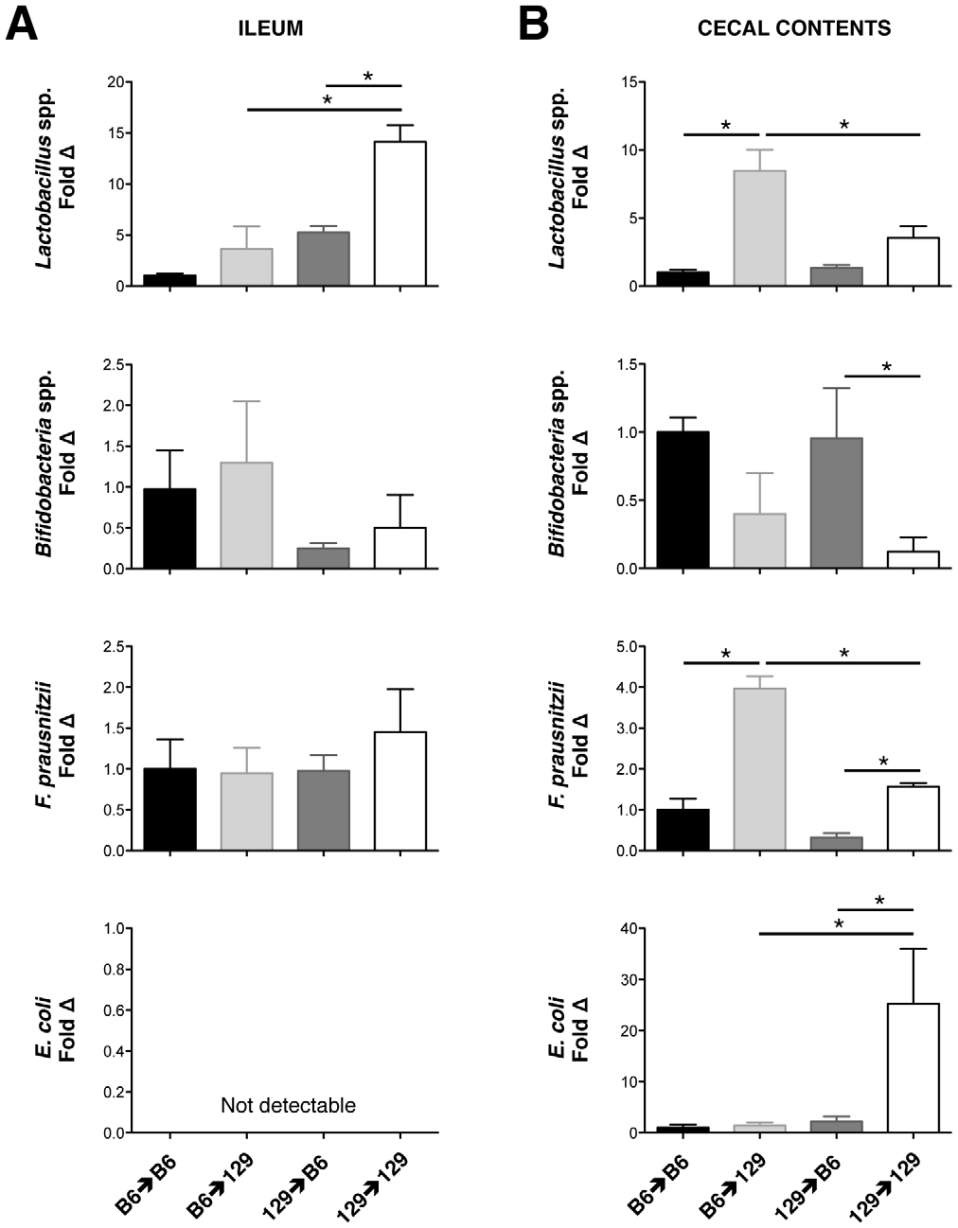
Host genotype and bacterial source affect abundances of specific bacterial groups within the intestinal microbiota. Quantification of specific bacterial groups from (A) ileal tissue samples, and (B) cecal contents using quantitative PCR of the 16 S rRNA gene is shown (n = 3–4 mice/group). Copy number is normalized to total 16 S, and fold change is relative to the B6 donor→B6 recipient group (black bars). Values are expressed as mean ± SEM (*P≤.05).

Because assessments of specific bacterial alterations between experimental groups cannot be made using T-RFLP, we next utilized directed qPCR of the 16 S rRNA gene to characterize specific bacterial differences between the enteric microbiota of B6 and 129 mice. Relevant bacterial groups were selected based on previously established roles as either “pro-inflammatory” or “protective” commensal organisms in regards to intestinal physiology and gut homeostasis [10]. The particular bacterial groups analyzed by qPCR included *E. coli* (pro-inflammatory), and *Lactobacillus* spp., *Bifidobacterium* spp., and *Faecalibacterium prausnitzii* (protective). In the mucosally-adherent ileal compartment, no differences in total bacteria numbers were observed across experimental groups (*P*<.69). Of the individual bacteria tested, only *Lactobacillus* showed significant differences (Fig. 5A). Specifically, when GF B6 and 129 mice both received identical 129 stool, higher ileal levels of *Lactobacillus* were ultimately detected in the 129 recipients, supporting a host genotype effect on *Lactobacillus* abundance. However, 129 recipients receiving stool from B6 donors developed lower levels of *Lactobacillus* than those receiving stool from 129 mice, suggesting an influence of the donor as well. This was confirmed by two-way ANOVA that identified both donor and recipient effects on final *Lactobacillus* levels (Table 1).

**Table 1:** Donor and recipient effects on specific bacterial group levels.

Bacterial Group	Donor Effect	Recipient Effect	Interaction [Donor×Recipient]
**ILEUM**			
*Lactobacillus* spp.	**0.0006**	**0.0034**	0.0634
*Bifidobacterium* spp.	0.1446	0.5672	0.9401
*F. prausnitzii*	0.5291	0.5727	0.4875
*E. coli*	n/a	n/a	n/a
**CECAL CONTENTS**			
*Lactobacillus* spp.	**0.0206**	**0.0002**	**0.0105**
*Bifidobacterium* spp.	0.4770	**0.0081**	0.6048
*F. prausnitzii*	**<0.0001**	**<0.0001**	**0.0024**
*E. coli*	**0.0212**	**0.0283**	**0.0332**

P-values calculated using 2-way analysis of variance are shown for ileal and cecal content bacterial groups indicated. Values were determined based on bacterial levels summarized in Figure 5. Statistically significant values are highlighted in bold.

The luminal bacteria of the ileum could not be assessed due to the paucity of ileal lumen contents in the majority of mice. However, data describing specific bacterial levels within the cecal lumen are shown in Figure 5B. Again, no differences were found in total bacterial levels across all experimental groups (*P*<.1). However, in contrast to the ileal mucosal compartment, numerous luminal bacteria subtypes displayed significant differences across experimental groups. In general, 129 recipient mice supported higher levels of all bacterial groups tested, except for *Bifidobacterium*, which was lower in these animals. *E. coli* displayed a unique pattern of abundance, with substantial levels detectable only in 129 mice receiving 129 stool. Two-way ANOVA revealed that *Lactobacillus*, *F. prausnitzii*, and *E. coli* levels were all affected by both the donor bacterial source and recipient genotype (Table 1). Similar to the ileal mucosal results, this highlights the importance of both donor and host genotype effects on the final composition of the intestinal microbiota.

### 129 Mouse α-Defensins Exhibit Diminished Antimicrobial Activity Against Pro-Inflammatory E. coli Strains

**Figure 6:**
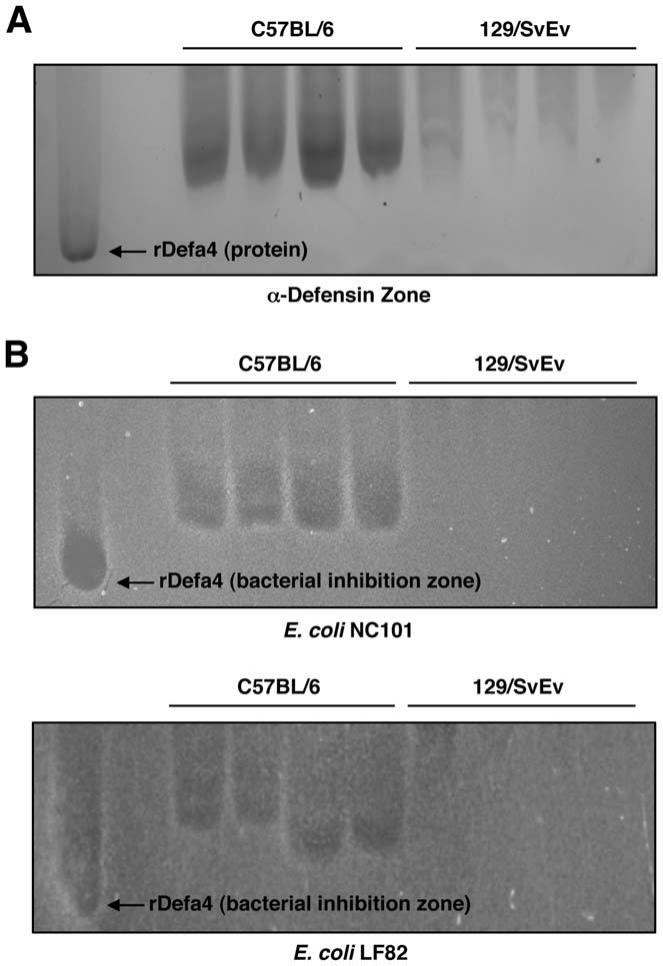
129 mouse α-defensins exhibit diminished antimicrobial activity against pro-inflammatory E. coli strains. (A) AU-PAGE was used to generate α-defensin profiles from B6 and 129 mice. The α-defensin region was excised and overlaid onto *E.coli*-laden agarose. (B) Zones of bacterial inhibition were detected for both NC101 (upper) and LF82 (lower) *E. coli* strains. Increased growth inhibition was consistently observed from α-defensins from B6 mice.

Numerous lines of evidence suggest that adherent-invasive *E. coli* (AIEC) subtypes may be pro-inflammatory in the context of chronic intestinal inflammatory conditions such as CD [22]. Specifically, the AIEC strains NC101 (murine origin) and LF82 (human CD origin) have both been shown to induce intestinal inflammation in genetically-engineered mice [23], [24]. Given the higher levels of *E. coli* observed in 129 mice, we hypothesized that ileal α-defensins isolated from 129 animals would have attenuated antimicrobial activity against *E. coli*, as compared to B6 mice. To test this hypothesis, we generated AU-PAGE gels similar to that shown previously in Figure 3B. We then excised the α-defensin zone (Fig. 6A), overlaid it onto an agarose plate of confluent *E. coli*, and observed for zones of bacterial inhibition. As shown in Figure 6B, the α-defensins extracted from B6 mice generated profound zones of bacterial inhibition, while those from 129 mice did not affect *E. coli* growth. This was true for both NC101 and LF82 *E. coli* strains. These findings putatively link the observed alterations in α-defensin expression to the changes in *E. coli* levels detected in 129 mice relative to B6 animals.

## Discussion

In this study, we describe key differences in Paneth cell function between B6 and 129 mouse strains. Specifically, 129 mice appear to have attenuated Paneth cell function relative to their B6 counterparts. In particular, 129 animals clearly possess fewer ileal Paneth cells than B6 mice. Although the precise determinants of intestinal Paneth cell numbers have yet to be elucidated, Wnt signaling (via β-catenin/TCF) plays a critical role for Paneth cell differentiation [25], likely through downstream molecules such as Eph receptors [26] and the SRY-box containing gene 9 [27], [28]. Recently, mice deficient in colony stimulating factor-1 and mice overexpressing the transcription factor caudal-related homeobox protein 2 were shown to have reduced numbers of Paneth cells [29], [30]. It is interesting to speculate that these molecules may be expressed at varying levels in different inbred mouse strains, and experiments are planned to examine this possibility.

Decreased Paneth cell numbers in 129 mice likely contribute to diminished overall Paneth cell function through the concomitant reduction of various AMPs such as Lyz and specific α-defensin isoforms (i.e. Defa3, Defa5). However, microbial factors also appear important. For example, *Ang4* transcripts were lower in 129 mice relative to B6 animals in the GF state, but similar between the 2 groups in the presence of an SPF microbiota. For *Reg3γ*, significant differences in expression were only apparent between B6 and 129 mice under SPF conditions. In both cases, the intestinal microbiota appears to play an important role in defining the relative levels of these AMPs. This is consistent with previous work describing differential AMP regulation through distinct antimicrobial expression programs [31].

Yet another contributor to the disparities of Paneth cell function between B6 and 129 mice are inherent differences of the AMP gene loci within these animals. Despite lower Paneth cell numbers, *Defa-rs10* was present in 129 mice, but undetectable in B6 animals. Our review of the NIH B6 mouse genome assembly reveals no evidence of the *Defa-rs10* gene in B6 animals. Similarly, mRNA for the AMP *Defa4* was undetectable in B6 mice, while *Defa20* was not found in 129 animals. This is consistent with previous reports that the B6 genome lacks the *Defa4* gene, while the Celera genome assembly (which includes various 129 strains) does not include the gene for *Defa20* [21].

The combination of altered Paneth cell numbers, variable microbial influences, and inherent AMP gene differences between B6 and 129 mice results in markedly different AMP expression patterns between these 2 mouse strains. In particular, α-defensin peptides display divergent expression profiles in B6 and 129 mice. Previous work has demonstrated that B6 mice have a unique AU-PAGE pattern of peptide migration [32], and a distinct profile of α-defensin peptides [21]. However, the present study represents the first reported characterization of α-defensin peptides from 129 mice, including a detailed analysis of individual α-defensin bands that has not been previously performed for any non-B6 strain. Specifically, peptides corresponding to Defa2/18v, Defa11, Defa16, and Defa18 are reported here for the first time. These data define specific differences in α-defensin expression between B6 and 129 mice, and expand the current repertoire of established α-defensin peptides.

In addition to variations of AMP expression between mouse strains, the present study also demonstrates the importance of host genetic background on the composition of the enteric microbiota. Several studies support the premise that host background strongly impacts the structure of the mouse gut microbiota [33]–[35]; however, these studies are limited by the strong influence that maternal microbial colonization exerts on the enteric microbiota of the offspring. Indeed, elegant work using implantation of genetically distinct mouse embryos into a single pseudo-pregnant mother revealed that the genetically distinct progeny had a similar gut microbial composition to the mother [36]. To determine the direct effects of host background strain on the composition of the enteric microbiota in adult mice, we utilized a novel approach of reciprocally colonizing GF mice of distinct background strains. Our results confirmed that adult B6 and 129 mouse strains indeed develop distinct ileal and fecal bacterial communities.

Although host genotype clearly plays a role in determining intestinal microbial composition, the importance of bacterial source should not be ignored. This influence is underscored by our analysis of specific bacterial groups within the intestinal microbiota of B6 versus 129 mouse recipients. These studies demonstrated that, in addition to recipient genotype, the donor bacterial source also played a substantial role in determining the ultimate levels of specific enteric bacterial groups. Specifically, ileal *Lactobacillus* levels and cecal content levels of *Lactobacillus*, *F. prausnitzii*, and *E. coli* were all influenced by both the donor and recipient strain. These findings highlight the importance of independent evaluation of specific bacteria, even when global microbial community profiling is performed. These data also suggest that despite the strong influence of host genetics, external manipulation of the intestinal microbiota is feasible. This will be essential if we are to develop treatment strategies based on modulation of the commensal microbiota.

While this study does not demonstrate that the enteric microbial alterations observed in B6 versus 129 mice are due directly to alterations in Paneth cell function, our findings suggest that Paneth cells may play a role in shaping gut microbial communities. This is consistent with previous work demonstrating profound changes in the gut microbiota of mice deficient in functional Paneth cell α-defensins [6]. Although we expected to find more bacterial differences in the ileum (due to the high abundance of Paneth cells in this location), previous work has demonstrated that functional Paneth cell antimicrobials can be detected in the colon [37]; therefore, increased cecal levels of the bacterial groups described in 129 mice are consistent with overall decreased AMP activity in these animals. This is further supported by our finding that 129 mice had diminished α-defensin antimicrobial activity against pro-inflammatory *E. coli* strains that were expanded in these animals. It was interesting to note that, in contrast to all other bacteria analyzed, *Bifidobacterium* spp. were decreased in 129 mice, with a very strong recipient effect (*P*<.008). This may be due to the fact that *Bifidobacteria* are highly fastidious organisms [38], and were thus potentially out-competed by more robust organisms that were able to thrive under conditions of attenuated Paneth cell activity in the 129 mice. Further studies are needed to test this theory.

Numerous potential biologic implications arise from the work described in this study, particularly in regards to the role of Paneth cells in modulating intestinal inflammation. B6 mice are relatively resistant to multiple models of acute and chronic intestinal inflammation, while 129 mice are highly colitogenic [39]–[41]. It is interesting to speculate that attenuated Paneth cell function may contribute to the increased susceptibility to intestinal inflammation observed in 129 mice. This is consistent with previous studies that suggest AMP dysfunction contributes to the development of experimental colitis. Mice with epithelial-specific ablation of Xbp1 [14] or IκB kinase-γ (NEMO) [19] have significant α-defensin deficiencies and increased susceptibility to colitis. Furthermore, Nod2^−/−^ mice develop granulomatous ileitis in the presence of *Helicobacter hepaticus*, a phenotype that is rescued when these animals are crossed with mice over-expressing a human α-defensin [42]. These studies highlight the importance of Paneth cells in the control of intestinal inflammation.

From a clinical standpoint, the findings reported in this study are particularly important in light of the emerging role of Paneth cells in the pathogenesis of CD. Indeed, ample evidence suggests that small intestinal CD may represent a complex disease of the Paneth cell [9]. Individuals with small intestinal CD have reduced ileal expression of Paneth cell α-defensins, a finding highly pronounced in patients with frameshift mutations of the NOD2 risk allele [12]. Paneth cell numbers in these patients were unaffected by inflammation [12], suggesting that diminished α-defensin levels were not due to solely Paneth cell loss. Patients with CD also have reduced expression and binding activity of TCF-4 [43], a Wnt pathway transcription factor known to regulate Paneth cell development and α-defensin expression [25]. Importantly, relatively minor changes in TCF expression result in profound attenuation of host antimicrobial function [43]. Interestingly, polymorphisms of the human TCF-4 promoter region are associated with small intestinal CD [15]. Additional CD risk polymorphisms including ATG16L1 and XBP1 have been linked to Paneth cell dysfunction [13], [14], implicating Paneth cells as important players in autophagy and the endoplasmic reticulum stress response [8]. The intersection of multiple key pathways involved in intestinal inflammation upon the Paneth cell emphasizes the potential importance of dysfunction of this cell type in CD pathogenesis.

In conclusion, the present study provides an in depth characterization of Paneth cell function and intestinal microbial composition in two commonly used inbred mouse strains. Our results support the important impact of host genotype on both Paneth cell activity and gut microbial composition. These findings establish a necessary baseline for future studies that may utilize these mouse strains to study Paneth cells and/or the enteric microbiota. Furthermore, they raise the interesting possibility that Paneth cells may be an important cellular player in a variety of complex medical disorders that have been linked to the intestinal microbiota, ranging from metabolic syndromes such as obesity and diabetes, to chronic intestinal inflammatory conditions such as CD. Using the foundation established in this study, future work may be directed towards establishing the genetic basis of the Paneth cell differences described. In particular, quantitative trait locus mapping of F_1_ and F_2_ mice derived from B6×129 crosses could be used to identify loci contributing to Paneth cell development and function. The identification of such loci may then lead to the genetic disruption of Paneth cell formation and function, providing an opportunity to directly determine the relative impact of Paneth cells on gut microbial composition and mouse phenotype.

## Materials and Methods

### Ethics Statement

All animals used in these studies were cared for in compliance with guidelines established by the American Association for Laboratory Animal Care and Research. All procedures were approved by the University of North Carolina at Chapel Hill Institutional Animal Care and Use Committee, which is accredited by the Association for the Assessment and Accreditation of Laboratory Animal Care.

### Mice

Adult, wild-type B6 (Jackson Laboratories, Bar Harbor, ME) and 129 (Taconic Laboratories, Germantown, NY) mice were housed in SPF conditions. All SPF mice were bred for multiple generations in the same animal room and sacrificed within 8–12 wk of age. Sterilely-derived B6 and 129 mice were maintained in GF conditions at the National Gnotobiotic Rodent Resource Center at the University of North Carolina at Chapel Hill, and colonized at 8 wk of age with the enteric microbiota of B6 or 129 mice raised in SPF conditions, as previously described [44]. Following colonization, each mouse group was housed separately for 12 wk prior to necropsy.

### Immunohistochemistry

2 cm segments of terminal ileum were fixed in 10% phosphate-buffered formalin for 12 hr. The tissue segments were longitudinally embedded in paraffin and cut in 5 µm sections for histological analysis. Sections were de-parafinized in separate containers of fresh xylene for a total of 8 min. Rehydration was accomplished in a series of ethanol dilutions. Sections were then treated with 3% hydrogen peroxide to inhibit the action of endogenous peroxidase, blocked with 3% bovine serum albumin (BSA) in phosphate buffered saline (PBS) for 1 h at 25°C, and incubated with 1∶1500 rabbit polyclonal antibody to human lysozyme (Diagnostic BioSystems, Pleasanton, CA) in 3% BSA overnight at 4°C. Sections were subsequently incubated with 1∶200 biotinylated anti-rabbit IgG (Vector Laboratories, Inc., Burlingame, CA) in 1% BSA for 30 min at 25°C, and a 1∶50 dilution of Avidin DH and biotinylated enzyme (Vector Laboratories, Inc.) in PBS for 30 min at 25°C. The sections were visualized with 3,3′-diaminobenzidine chromogen reagent (Dako, Carpinteria, CA).

### Flow Cytometry

#### 1. Epithelial cell isolation

Ileal epithelial cells were isolated using ethylenediaminetetraacetic acid (EDTA)/dispase digestion as previously described [45]. Briefly, mouse ilea were flushed with cold PBS, opened lengthwise, and bathed over ice in PBS/30 mM EDTA/1.5 mM dithiothreitol for 20 min. The tissue was then shaken in fresh PBS/30 mM EDTA for 30 s followed by incubation at 37°C for 10 min. Dissociated crypts and villi were then pelleted at 2,500 revolution/min for 5 min. The cells were washed with PBS, and re-suspended in Hank's Balanced Salt Solution (HBSS)/0.3 U/ml dispase at 37°C. Shaking was performed every 2 min for 10 min. 100 µg DNaseI was then added, and the cells were passed through a 70-µm filter. Cells were pelleted at 2,500 revolution/min for 5 min and re-suspended in 4 ml HBSS with 5% FBS, followed by passage through a 100-µm filter and combination with an additional 100 µg DNaseI.

#### 2. Flow cytometry analysis

Cells were fixed in 4% paraformaldehyde for 15 min, washed with PBS, and re-suspended in saponin permeabilization buffer (Invitrogen, Carlsbad, CA) with Lyz-fluorescein isothiocyanate antibody (1∶10, Dako, Carpinteria, CA) at room temperature (RT) for 30 min. All flow analyses were performed using a CyAn Flow Cytometer (Dako/Cytomation). Doublets were excluded using a bivariate plot of pulse width vs. forward scatter. Hematopoietic cell exclusion was accomplished using side-scatter/forward-scatter gating that had previously been optimized to exclude all CD45^+^ cells from identical epithelial preparations [46].

### Real-Time Reverse-Transcriptase Polymerase Chain Reaction

Total RNA was extracted from ileal tissue using an RNeasy Mini Kit (Qiagen, Valencia, CA) per manufacturer's instructions. Complimentary DNA was generated using SuperScript II reverse transcriptase (Invitrogen, Carlsbad, CA). Quantitative RT-PCR was performed using TaqMan Gene Expression Master Mix (Applied Biosystems, Foster City, CA) per manufacturer's instructions. Specific primer/probe sets were obtained from Applied Biosystems as follows: *Gapdh* (Mm99999915_g1), *Lyz* (Mm00727183_s1), *Ang4* (Mm03647554_g1), *Reg3γ* (Mm00441127_m1), *Defa3* (Mm04205962_gH), *Defa5* (Mm00651548_g1), *Defa4* (Mm00651736_g1), *Defa20* (Mm00842045g1), and *Defa-rs10* (Mm00833275_g1). Global α-defensin primers were: forward, 5′-GGTGATCATCAGACCCCAGCATCAGT-3′; reverse, 5′-AAGAGACTAAAACTGAGGA GCAGC-3′. These were amplified using SYBR Green PCR Master Mix (Applied Biosystems), per manufacturer's protocols. *Gapdh* was used as an internal control, and ΔΔCt values were calculated to obtain fold changes relative to the baseline group.

### Acid Urea Polyacrylamide Gel Electrophoresis

Samples were subject to 2 protein extraction steps using 60% acetonitrile, 1% trifluoroacetic acid (TFA), incubated at 4°C with rotation and clarification by centrifugation. Resulting supernatants were lyophilized, re-suspended and dialyzed in 5% acetic acid. Dialysates were then lyophilized, and 300 µg protein aliquots were solubilized in 30 µl of AU-PAGE loading solution (3 M urea, 5% acetic acid). These were next electrophoresed on a 12.5% AU-PAGE gel for 1 h at 150 V and 4 h at 400 V. All extracts were analyzed alongside a sample of recombinant α-defensin 4 (rDefa4) that was kindly provided by Dr. André Ouellette of the University of Southern California. Resolved proteins were then visualized by staining with 0.05% Coomassie Brilliant Blue in 30% methanol and 15% formalin, followed by destaining in 25% methanol and 1% formalin.

### Mass Spectrometry Analysis

Individual Coomassie-stained AU-PAGE bands were excised and de-stained twice in 50% acetonitrile, 25 mM ammonium bicarbonate at room temperature for 10 min. Lyophilized gel slices were extracted for protein with 0.5% TFA, 50% acetonitrile in two steps at room temperature for 10 min. Extracted protein was subsequently lyophilized and resuspended in 0.1% TFA for analysis. Samples were submitted to the Proteomics Core Facility of the University of North Carolina at Chapel Hill School of Medicine for mass spectrometric analysis via MALDI-TOF MS/MS in the linear mode using α-cyano-4-hydrocinnamic acid for the matrix.

### Bacterial Composition Analyses

#### 1. Bacterial DNA extraction

100 mg of flushed, frozen tissue (to assess mucosally-associated bacteria) or feces/cecal contents (to assess luminal bacteria) was re-suspended in sterile lysis buffer (200 mM NaCl, 100 mM Tris [pH 8.0], 20 mM EDTA, 20 mg/ml Lyz [Sigma-Aldrich, St. Louis, MO]) for 30 min at 37°C. This was then supplemented with 40 µl of proteinase K (20 mg/ml) and 85 µl of 10% SDS and incubated for 30 min at 65°C. Homogenization was accomplished by adding 300 mg of 0.1 mm zirconium beads (BioSpec Products, Bartlesville, OK) and bead beating for 2 min (BioSpec). Supernatants were then isolated and DNA extracted using phenol/chloroform/iso-amyl alcohol (25∶24∶1), followed by precipitation with absolute ethanol for 1 hr at −20°C. Finally, the precipitated DNA was cleaned up using a DNeasy Blood and Tissue extraction kit (Qiagen) per manufacturer's instructions.

#### 2. Terminal restriction fragment length polymorphism analysis

The 16 S ribosomal RNA gene was amplified by PCR using fluorescently labeled primers (forward primer 8F FAM 5′-AGAGTTTGATCCTGGCTCAG-3′ and reverse primer 1492R Hex 5′-GGTTACCTTGTTACGACTT-3′). Amplicons were then purified using a Qiagen PCR purification kit, followed by digestion with *Hha* I to generate terminal restriction fragments (TRFs) of varying size. The TRFs were processed by capillary electrophoresis on an ABI 3100 genetic analyzer. Genemapper software (Applied Biosystems) was used to determine the size and height of each TRF. The size of each TRF corresponded to a different bacterium or bacterial group due to polymorphisms in the 16 S rRNA gene. Size and abundance data were compiled into a data matrix using Sequentix software (Sequentix, Germany). Further analysis is described in the “Statistical Analysis” section below.

#### 3. Quantitative 16 S PCR

Quantitative PCR was accomplished using primers for the 16 S rRNA gene of specific bacterial groups as follows: Total bacteria (forward, 5′-GTGSTGCAYGGYTGTCGTCA-3′ and reverse, 5′-ACGTCRTCCMCACCTTCCTC-3′); *Lactobacillus* spp. (forward 5′-AGCAGTAGGGAATCTCCA-3′ and reverse, 5′-CACCGCTACACATGGAG-3′); *Bifidobacterium* spp. (forward, 5′-GGGTGGTAATGCCGGATG-3′ and reverse, 5′-CACCGCTACACATGGAG-3′); *F. prausnitzii* (forward, 5′-GATGGCCTCGCGTCCGATTAG-3′ and reverse, 5′-CCGAAGACCTTCTTCCTCC-3′); *E. coli* (forward, 5′-GTTAATACCTTTGCTCATTGA-3′ and reverse, 5′-ACCAGGGTATCTAATCCTGTT-3′). All assays were performed in 96-well plates using an Eppendorf Realplex^2^ mastercycler thermocycler (Eppendorf, Hauppauge, NY). Each reaction was carried out in 25 µl volumes containing 1× SYBR Green PCR master mix (Applied Biosystems), 0.5 µM of each primer, and 50–100 ng of DNA. Melting curves were assessed to ensure specificity of PCR products, and all plates include a “no template” negative control. PCR conditions were: 10 min at 95°C, followed by 40 cycles of 95°C for 15 s, 20 s at 50°C, and 72°C for 1 min. Quantitative PCR standards were created by amplifying and cloning the target 16 S rRNA genes from appropriate positive control strains. Standard curves were then generated for each bacterial group and used to enumerate copy number in individual samples. Bacterial levels were calculated as a percentage relative to total bacteria 16 S copy number, normalized to the baseline B6→B6 group, and expressed as a mean fold change ± standard error of the mean (SEM).

### Bactericidal Gel Overlay Assay

*E. coli* strains NC101 [23] and LF82 [24] were grown to mid-log phase in trypticase soy broth (TSB) media, washed with 10 mM sodium citrate-phosphate buffer, re-suspended in warm 0.03% TSB, 1% low-melt agarose, 10 mM sodium citrate-phosphate buffer and 0.02% Tween 20 at 4×10^5^ CFU/mL, and plated onto a Petri dish as a 1 mm deep undergel. Ileal protein samples (100 µg) were prepared by electrophoresis on a small-scale 12.5% AU-PAGE gel for 1 h at 150V. Resolved gels were washed with ice-cold 10 mM sodium phosphate buffer for 15 min, placed atop the bacteria-laden solid agarose layer, and incubated at 37°C for 3 h. Subsequently, the gel was removed and replaced with a layer of warm 6% TSB, 0.8% low-melt agarose to form a nutrient-rich overgel. Gel overlay plates were incubated overnight at 37°C and then imaged for band-associated zones of bacterial clearance.

### Statistical Analysis

#### 1. AMP expression analysis

All analyses were performed using the GraphPad Prism 5 software suite (GraphPad, San Diego, CA). For all variables of interest, distribution was assessed and found to be normal unless otherwise stated. Means of normally distributed data were compared using one-way analysis of variance (ANOVA), followed by Tukey's multiple comparison testing and 2-tailed, unpaired Student's *t* tests. When data was not normally distributed, the Kruskal-Wallis test was utilized to compare means, followed by Dunn's multiple comparison testing and 2-tailed Mann-Whitney tests. Data are expressed as mean ± SEM.

#### 2. T-RFLP analysis

All TRF data were standardized (individual TRF peak height as a proportion of total TRF peak heights within that sample), transformed by square root, and compiled into a Bray Curtis similarity matrix using PRIMER v. 6 (Primer-e, Ivybridge, UK). To test for differences in global community composition, TRF data were subjected to hierarchical cluster followed by analysis of similarity (ANOSIM) to generate relevant P-values. Multidimensional scaling plots were constructed to illustrate similarity/dissimilarity. Biodiversity of each sample was assessed by Shannon-Weiner diversity index; differences in richness or diversity between treatment groups were assessed by Student's *t* test. Similarity percentages (SIMPER) was used to determine which TRFs contributed most to similarity within a group, after a 10% cutoff (stool samples) for low contributors.

#### 3. 16 S qPCR analysis

Means were analyzed as described above for AMP expression. To determine effects of donor and recipient genotype on bacterial composition, two-by-two donor versus recipient tables were generated using qPCR data for each bacterial group, and subjected to two-way ANOVA, with the α set at *P*<.05.

## Supporting Information

Table S1 **C57BL/6 and 129/SvEv mice express distinct enteric α-defensins at the peptide-level.** Observed mass-to-charge (m/z) values of prominent, software-indicated mass peaks found within the expected cryptdin mass range (2499.0 to 4518.0 m/z) are tabulated in correspondence to the gel bands labeled in Fig. 3B. The observed m/z values are listed in increasing value and are classified as being major or minor based on whether the signal intensities of the mass peaks were greater or lesser than 50% of signal intensity relative to baseline, respectively. Determination of the cryptdin identity of individual m/z values was assisted by a comparison of the observed m/z values with the calculated m/z values of all purified and transcript- and gene-predicted cryptdin peptides based upon their oxidized, singly-protonated forms. Matches of m/z values with greater than 99.8% identity or with mass differences less than 5 Da were deemed sufficient for individual identification of mass peaks. Percent identity of m/z values was calculated from the quotient of the lesser over the greater m/z values of matched pairs. The label vDefa24 is used for various non-identical masses since there are multiple Defa24 variant transcripts that have yet to acquire unambiguous identifiers. To indicate a non-identifiable m/z value, the label N.I. was used. Note that the identification of Defa20 was extrapolated by the presence of an m/z value that was more than 98% identical to the predicted m/z value of the doubly-protonated form of oxidized Defa20, since mass spectrometric analysis was performed outside the mass range of its predicted, singly-protonated-based m/z value (4950.69).
(DOC)

## References

[1] Sato T, van Es JH, Snippert HJ, Stange DE, Vries RG (2011). Paneth cells constitute the niche for Lgr5 stem cells in intestinal crypts.. Nature.

[2] Ouellette AJ (2010). Paneth cells and innate mucosal immunity.. Curr Opin Gastroenterol.

[3] Ayabe T, Satchell DP, Wilson CL, Parks WC, Selsted ME (2000). Secretion of microbicidal alpha-defensins by intestinal Paneth cells in response to bacteria.. Nat Immunol.

[4] Wilson CL, Ouellette AJ, Satchell DP, Ayabe T, Lopez-Boado YS (1999). Regulation of intestinal alpha-defensin activation by the metalloproteinase matrilysin in innate host defense.. Science.

[5] Brandl K, Plitas G, Schnabl B, DeMatteo RP, Pamer EG (2007). MyD88-mediated signals induce the bactericidal lectin RegIII gamma and protect mice against intestinal Listeria monocytogenes infection.. J Exp Med.

[6] Salzman NH, Hung K, Haribhai D, Chu H, Karlsson-Sjoberg J (2010). Enteric defensins are essential regulators of intestinal microbial ecology.. Nat Immunol.

[7] Backhed F, Ley RE, Sonnenburg JL, Peterson DA, Gordon JI (2005). Host-bacterial mutualism in the human intestine.. Science.

[8] Kaser A, Blumberg RS (2011). Autophagy, microbial sensing, endoplasmic reticulum stress, and epithelial function in inflammatory bowel disease.. Gastroenterology.

[9] Wehkamp J, Stange EF (2010). Paneth's disease.. J Crohns Colitis.

[10] Sartor RB (2008). Microbial influences in inflammatory bowel diseases.. Gastroenterology.

[11] Keshav S (2006). Paneth cells: leukocyte-like mediators of innate immunity in the intestine.. J Leukoc Biol.

[12] Wehkamp J, Harder J, Weichenthal M, Schwab M, Schaffeler E (2004). NOD2 (CARD15) mutations in Crohn's disease are associated with diminished mucosal alpha-defensin expression.. Gut.

[13] Cadwell K, Liu JY, Brown SL, Miyoshi H, Loh J (2008). A key role for autophagy and the autophagy gene Atg16l1 in mouse and human intestinal Paneth cells.. Nature.

[14] Kaser A, Lee AH, Franke A, Glickman JN, Zeissig S (2008). XBP1 links ER stress to intestinal inflammation and confers genetic risk for human inflammatory bowel disease.. Cell.

[15] Koslowski MJ, Kubler I, Chamaillard M, Schaeffeler E, Reinisch W (2009). Genetic variants of Wnt transcription factor TCF-4 (TCF7L2) putative promoter region are associated with small intestinal Crohn's disease.. PLoS One.

[16] Hooper LV, Stappenbeck TS, Hong CV, Gordon JI (2003). Angiogenins: a new class of microbicidal proteins involved in innate immunity.. Nat Immunol.

[17] Cash HL, Whitham CV, Behrendt CL, Hooper LV (2006). Symbiotic bacteria direct expression of an intestinal bactericidal lectin.. Science.

[18] Bevins CL, Salzman NH (2011). Paneth cells, antimicrobial peptides and maintenance of intestinal homeostasis.. Nat Rev Microbiol.

[19] Nenci A, Becker C, Wullaert A, Gareus R, van Loo G (2007). Epithelial NEMO links innate immunity to chronic intestinal inflammation.. Nature.

[20] Cash HL, Whitham CV, Behrendt CL, Hooper LV (2006). Symbiotic bacteria direct expression of an intestinal bactericidal lectin.. Science.

[21] Shanahan MT, Tanabe H, Ouellette AJ (2011). Strain-specific polymorphisms in Paneth cell a-defensins of C57BL/6 mice and evidence of vestigial myeloid a-defensin pseudogenes.. Infect Immun.

[22] Rolhion N, Darfeuille-Michaud A (2007). Adherent-invasive Escherichia coli in inflammatory bowel disease.. Inflamm Bowel Dis.

[23] Kim SC, Tonkonogy SL, Albright CA, Tsang J, Balish EJ (2005). Variable phenotypes of enterocolitis in interleukin 10-deficient mice monoassociated with two different commensal bacteria.. Gastroenterology.

[24] Carvalho FA, Barnich N, Sivignon A, Darcha C, Chan CHF (2009). Crohn's disease adherent-invasive Escherichia coli colonize and induce strong gut inflammation in transgenic mice expressing human CEACAM.. J Exp Med.

[25] van Es JH, Jay P, Gregorieff A, van Gijn ME, Jonkheer S (2005). Wnt signalling induces maturation of Paneth cells in intestinal crypts.. Nat Cell Biol.

[26] Batlle E, Henderson JT, Beghtel H, van den Born MMW, Sancho E (2002). Beta-catenin and TCF mediate cell positioning in the intestinal epithelium by controlling the expression of EphB/ephrinB.. Cell.

[27] Mori-Akiyama Y, van den Born M, van Es JH, Hamilton SR, Adams HP (2007). SOX9 is required for the differentiation of paneth cells in the intestinal epithelium.. Gastroenterology.

[28] Bastide P, Darido C, Pannequin J, Kist R, Robine S (2007). Sox9 regulates cell proliferation and is required for Paneth cell differentiation in the intestinal epithelium.. J Cell Biol.

[29] Huynh D, Dai XM, Nandi S, Lightowler S, Trivett M (2009). Colony stimulating factor-1 dependence of paneth cell development in the mouse small intestine.. Gastroenterology.

[30] Crissey MAS, Guo R-J, Funakoshi S, Kong J, Liu J (2011). Cdx2 levels modulate intestinal epithelium maturity and Paneth cell development.. Gastroenterology.

[31] Vaishnava S, Behrendt CL, Ismail AS, Eckmann L, Hooper LV (2008). Paneth cells directly sense gut commensals and maintain homeostasis at the intestinal host-microbial interface.. Proc Natl Acad Sci U S A.

[32] Shirafuji Y, Tanabe H, Satchell DP, Henschen-Edman A, Wilson CL (2003). Structural determinants of procryptdin recognition and cleavage by matrix metalloproteinase-7.. J Biol Chem.

[33] Esworthy RS, Smith DD, Chu FF (2010). A Strong Impact of Genetic Background on Gut Microflora in Mice.. Int J Inflam.

[34] Benson AK, Kelly SA, Legge R, Ma F, Low SJ (2010). Individuality in gut microbiota composition is a complex polygenic trait shaped by multiple environmental and host genetic factors.. Proc Natl Acad Sci U S A.

[35] Kovacs A, Ben-Jacob N, Tayem H, Halperin E, Iraqi FA (2011). Genotype is a stronger determinant than sex of the mouse gut microbiota.. Microb Ecol.

[36] Friswell MK, Gika H, Stratford IJ, Theodoridis G, Telfer B (2010). Site and strain-specific variation in gut microbiota profiles and metabolism in experimental mice.. PLoS One.

[37] Mastroianni JR, Ouellette AJ (2009). Alpha-defensins in enteric innate immunity: functional Paneth cell alpha-defensins in mouse colonic lumen.. J Biol Chem.

[38] Reuter G (2001). The Lactobacillus and Bifidobacterium microflora of the human intestine: composition and succession.. Curr Issues Intest Microbiol.

[39] Berg DJ, Davidson N, Kuhn R, Muller W, Menon S (1996). Enterocolitis and colon cancer in interleukin-10-deficient mice are associated with aberrant cytokine production and CD4(+) TH1-like responses.. J Clin Invest.

[40] Pena JA, Thompson-Snipes L, Calkins PR, Tatevian N, Puppi M (2009). Alterations in myeloid dendritic cell innate immune responses in the Galphai2-deficient mouse model of colitis.. Inflamm Bowel Dis.

[41] Mahler M, Bristol IJ, Sundberg JP, Churchill GA, Birkenmeier EH (1999). Genetic analysis of susceptibility to dextran sulfate sodium-induced colitis in mice.. Genomics.

[42] Biswas A, Liu Y-J, Hao L, Mizoguchi A, Salzman NH (2010). Induction and rescue of Nod2-dependent Th1-driven granulomatous inflammation of the ileum.. Proc Natl Acad Sci U S A.

[43] Wehkamp J, Wang G, Kubler I, Nuding S, Gregorieff A (2007). The Paneth cell alpha-defensin deficiency of ileal Crohn's disease is linked to Wnt/Tcf-4.. J Immunol.

[44] Sellon RK, Tonkonogy S, Schultz M, Dieleman LA, Grenther W (1998). Resident enteric bacteria are necessary for development of spontaneous colitis and immune system activation in interleukin-10-deficient mice.. Infect Immun.

[45] Formeister EJ, Sionas AL, Lorance DK, Barkley CL, Lee GH (2009). Distinct SOX9 levels differentially mark stem/progenitor populations and enteroendocrine cells of the small intestine epithelium.. Am J Physiol Gastrointest Liver Physiol.

[46] von Furstenberg RJ, Gulati AS, Baxi A, Doherty JM, Stappenbeck TS (2011). Sorting mouse jejunal epithelial cells with CD24 yields a population with characteristics of intestinal stem cells.. Am J Physiol Gastrointest Liver Physiol.

