# Supplementary material for: Mouse Background Strain Profoundly Influences Paneth Cell Function and Intestinal Microbial Composition
Source: PLoS One. 2012 Feb 27;7(2):e32403. doi: 10.1371/journal.pone.0032403 (PMC3288091; doi:10.1371/journal.pone.0032403)
Supplement: Table S1 — C57BL/6 and 129/SvEv mice express distinct enteric α-defensins at the peptide-level. Observed mass-to-charge (m/z) values of prominent, software-indicated mass peaks found within the expected cryptdin mass range (2499.0 to 4518.0 m/z) are tabulated in correspondence to the gel bands labeled in Fig. 3B. The observed m/z values are listed in increasing value and are classified as being major or minor based on whether the signal intensities of the mass peaks were greater or lesser than 50% of signal intensity relative to baseline, respectively. Determination of the cryptdin identity of individual m/z values was assisted by a comparison of the observed m/z values with the calculated m/z values of all purified and transcript- and gene-predicted cryptdin peptides based upon their oxidized, singly-protonated forms. Matches of m/z values with greater than 99.8% identity or with mass differences less than 5 Da were deemed sufficient for individual identification of mass peaks. Percent identity of m/z values was calculated from the quotient of the lesser over the greater m/z values of matched pairs. The label vDefa24 is used for various non-identical masses since there are multiple Defa24 variant transcripts that have yet to acquire unambiguous identifiers. To indicate a non-identifiable m/z value, the label N.I. was used. Note that the identification of Defa20 was extrapolated by the presence of an m/z value that was more than 98% identical to the predicted m/z value of the doubly-protonated form of oxidized Defa20, since mass spectrometric analysis was performed outside the mass range of its predicted, singly-protonated-based m/z value (4950.69). (DOC) [file pone.0032403.s001.doc]

**Table S1.**

| **AU-PAGE Band** | **Mass Peak Prominence** | **Observed m/z** | **Identified α-defensin** | **Theoretical m/z** | **Percent Identity** |
| --- | --- | --- | --- | --- | --- |
| **C57BL/6** |  |  |  |  |  |
| 1 | Major | 4317.1831 | Defa5 | 4316.20 | 99.98% |
|  | *Minor* | 4371.1211 | N.I. |  |  |
| 2 | Major | 4078.4815 | vDefa24-N | 4079.90 | 99.97% |
|  | Major | 4094.1155 | vDefa24 | 4094.90 | 99.98% |
|  | Major | 4130.3325 | Defa16 | 4134.92 | 99.89% |
|  | *Minor* | 4062.7605 | Defa24 | 4061.87 | 99.98% |
|  | *Minor* | 4164.2817 | vDefa2,18-N | 4160.00 | 99.90% |
|  | *Minor* | 4182.8081 | vDefa24 | 4179.85 | 99.93% |
| 3 | Major | 2503.2424 | Defa20***** | 2475.34 | 98.88% |
|  | Major | 4246.6167 | Defa2 | 4248.08 | 99.97% |
|  | Major | 4307.5488 | vDefa3 | 4308.01 | 99.99% |
|  | Major | 4330.9966 | Defa21 | 4330.11 | 99.98% |
|  | *Minor* | 4231.6104 | vDefa23 | 4231.04 | 99.99% |
|  | *Minor* | 4383.6895 | N.I. |  |  |
| 4 | Major | 4247.0679 | Defa2 | 4248.08 | 99.98% |
|  | Major | 4293.8032 | N.I. |  |  |
|  | Major | 4308.0107 | vDefa3 | 4308.01 | 100.00% |
|  | Major | 4345.7598 | Defa22 | 4344.19 | 99.96% |
|  | *Minor* | 4189.4033 | vDefa24 | 4193.81 | 99.89% |
|  | *Minor* | 4232.1138 | vDefa23 | 4231.04 | 99.97% |
|  | *Minor* | 4361.4146 | N.I. |  |  |
|  | *Minor* | 4396.5571 | N.I. |  |  |
| 5 | Major | 4346.0547 | Defa22 | 4344.19 | 99.96% |
|  | *Minor* | 4399.7944 | N.I. |  |  |
| **rDefa4** |  |  |  |  |  |
| 6 | Major | 3758.9697 | Defa4 | 3756.51 | 99.93% |
| **129/SvEv** |  |  |  |  |  |
| 7 | Major | 4317.0503 | Defa5 | 4316.25 | 99.98% |
|  | *Minor* | 4369.5933 | N.I. |  |  |
| 8 | Major | 4133.0396 | Defa6 | 4130.98 | 99.95% |
|  | Major | 4147.4951 | Defa11 | 4146.98 | 99.99% |
|  | Major | 4163.4805 | vDefa2,18-N | 4160.00 | 99.92% |
|  | Major | 4200.9448 | N.I. |  |  |
|  | Major | 4266.5708 | Defa25 | 4267.10 | 99.99% |
|  | Major | 4280.3462 | Defa18 | 4278.17 | 99.95% |
|  | *Minor* | 4118.5532 | Defa1 | 4116.89 | 99.96% |
|  | *Minor* | 4307.7178 | vDefa3 | 4308.01 | 99.99% |
|  | *Minor* | 4333.4004 | Defa21 | 4330.11 | 99.92% |
| 9 | Major | 4264.8403 | vDefa2-C | 4262.11 | 99.94% |
|  | Major | 4281.3281 | Defa18 | 4278.17 | 99.93% |
|  | Major | 4308.0762 | vDefa3 | 4308.01 | 100.00% |
|  | *Minor* | 3757.6594 | Defa4 | 3756.51 | 99.97% |
| 10 | Major | 3757.6768 | Defa4 | 3756.51 | 99.97% |
